# Stress-Mediated *cis*-Element Transcription Factor Interactions Interconnecting Primary and Specialized Metabolism *in planta*

**DOI:** 10.3389/fpls.2016.01725

**Published:** 2016-11-25

**Authors:** S. A. Sheshadri, M. J. Nishanth, Bindu Simon

**Affiliations:** School of Chemical and Biotechnology, SASTRA UniversityThanjavur, India

**Keywords:** stress, c*is*-elements, primary metabolism, specialized metabolism, transcriptional regulation

## Abstract

Plant specialized metabolites are being used worldwide as therapeutic agents against several diseases. Since the precursors for specialized metabolites come through primary metabolism, extensive investigations have been carried out to understand the detailed connection between primary and specialized metabolism at various levels. Stress regulates the expression of primary and specialized metabolism genes at the transcriptional level *via* transcription factors binding to specific *cis*-elements. The presence of varied *cis*-element signatures upstream to different stress-responsive genes and their transcription factor binding patterns provide a prospective molecular link among diverse metabolic pathways. The pattern of occurrence of these *cis*-elements (overrepresentation/common) decipher the mechanism of stress-responsive upregulation of downstream genes, simultaneously forming a molecular bridge between primary and specialized metabolisms. Though many studies have been conducted on the transcriptional regulation of stress-mediated primary or specialized metabolism genes, but not much data is available with regard to *cis*-element signatures and transcription factors that simultaneously modulate both pathway genes. Hence, our major focus would be to present a comprehensive analysis of the stress-mediated interconnection between primary and specialized metabolism genes *via* the interaction between different transcription factors and their corresponding *cis*-elements. In future, this study could be further utilized for the overexpression of the specific transcription factors that upregulate both primary and specialized metabolism, thereby simultaneously improving the yield and therapeutic content of plants.

## Introduction

Plants produce a wide array of biomolecules through metabolic pathways that are essential for sustenance of life. All the processes involved in plant primary metabolism are essential for maintenance of plant life and growth, whereas compounds resulting from specialized metabolism (specialized metabolites) have a role in plant defense and are also used as therapeutics in human disease treatment. Although primary and specialized metabolic processes are intimately interconnected, with the former providing precursors to the latter, yet most of the specialized metabolism processes have been studied largely in isolation and relatively little is known about their integration with primary metabolism (Tohge et al., 2013; Caretto et al., 2015).

**Figure 1:**
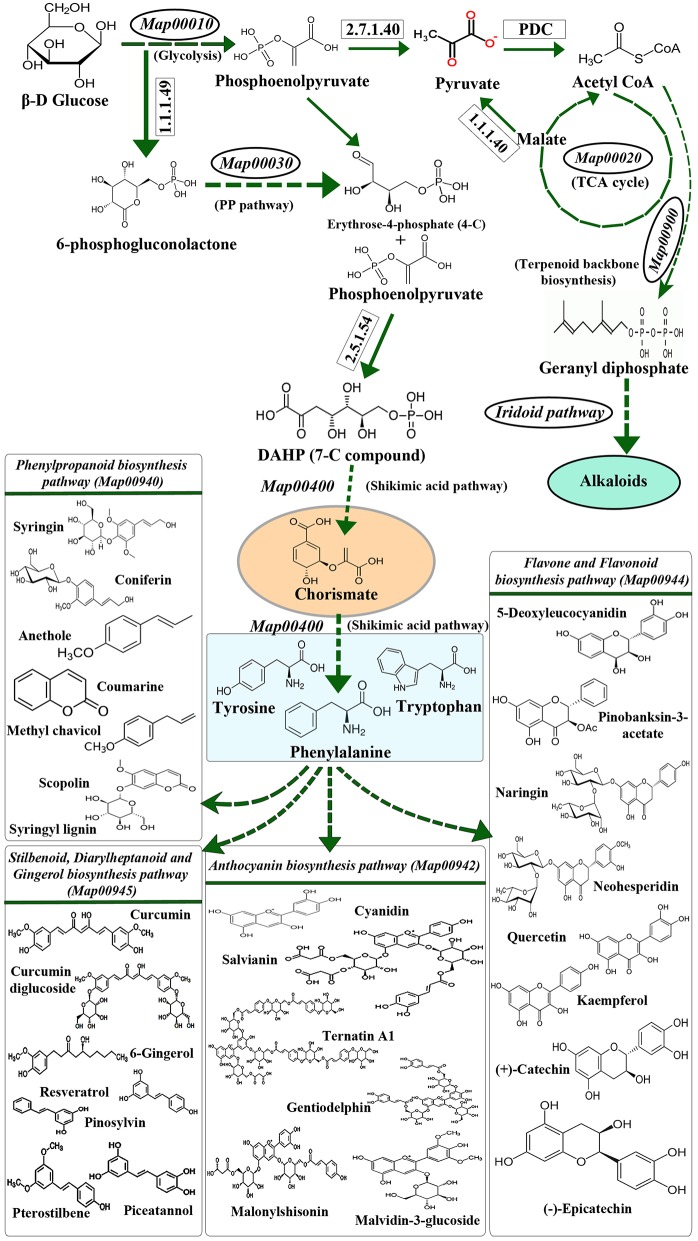
**The biochemical link between primary and specialized metabolism**. Primary and major specialized metabolisms (example, phenylpropanoid metabolism) are interconnected through intermediates like chorismate. The precursors for the synthesis of amino acids phenylalanine, tyrosine and tryptophan are derived through the Shikimic acid pathway and utilized in the biosynthesis of various specialized metabolites *via* the Phenylpropanoid biosynthesis pathway. (Map numbers indicate the KEGG pathway ID; PP pathway refers to Pentose phosphate pathway; PDC refers to Pyruvate Dehydrogenase Complex).

The extensive interrelationship between primary and specialized metabolism is a combined consequence of metabolite partitioning, energy donation and molecular signaling (Ibrahim and Jaafar, 2012). Principal primary metabolic pathways like Pentose Phosphate Pathway, TCA cycle, Photosynthesis, Glycolysis, etc. contribute to these intermediate metabolites, which act as precursors for specialized metabolic processes (Caretto et al., 2015; KEGG Map01100, Figure 1). The levels of these intermediates in their respective pools is governed by various physiological and genetic factors, like environmental stress, location of the system, inherited mutations, etc. (Tohge et al., 2013). Among all, environmental stress acts as a common mediator toward simultaneous upregulation of many primary and specialized metabolic pathways in plants (Bhargava and Sawant, 2013; Schlüter et al., 2013). It is also known to influence primary metabolic pathways like Carbon, Nitrogen and Phosphorous metabolism, as well as specialized metabolic pathways like Phenylpropanoid and Indole Alkaloid biosynthesis (Bhargava and Sawant, 2013; Schlüter et al., 2013; Rejeb et al., 2014), thereby causing upregulation of cascade of stress-responsive genes which impart stress-tolerance to the plants (Gao et al., 1998; Shulze et al., 2005; Ramakrishna and Ravishankar, 2011; Bhargava and Sawant, 2013; Schlüter et al., 2013; Gujjar et al., 2014; Caretto et al., 2015; Le Gall et al., 2015). Under stressed conditions, molecular level changes occurring in plants are principally brought about by transcription factors (TF) binding to their specific recognition sequences upstream to the stress-responsive genes (called as *cis*-elements). Although exhaustive data is available pertaining to the broad effects of the stress mediated primary and specialized metabolism (Bhargava and Sawant, 2013; Caretto et al., 2015), not many reports highlight the plausible role of *cis*-element and TF interactions in simultaneous regulation of primary and specialized metabolism genes.

The aim of this article is to unravel the interconnection between primary and specialized metabolism under various stress conditions, especially at the transcriptional level. As a part of our study, we have shown the effects of different stress on metabolites and genes specific to primary and specialized metabolic pathways. Further, we present an in-depth analysis of the stress-mediated primary and specialized metabolism genes with regard to their *cis*-element and TF interactions. To conclude, a detailed study is presented on the TFs that might play a role in simultaneous upregulation of primary and specialized metabolism genes.

## Stress conditions regulating primary and specialized metabolism genes

Plant systems are prone to a wide spectrum of stress conditions, like drought, salinity, temperature extremities, heavy metals, biotic (pathogen attacks) and human factors (herbicides, pesticides, weedicides, pollution, loss of gene pool) (Yadav, 2010). As a consequence, an estimated average global crop loss of 50% is caused due to varied stress conditions (Grover et al., 1998; Peleg et al., 2011; Haggag et al., 2015). Farmers additionally face numerous problems, including erratic and scanty rainfall, saline/alkaline soils, flash floods, water logging and global warming, which basically act as environmental stress, thereby hampering the overall productivity (Jenks and Hasegawa, 2005).

**Figure 2:**
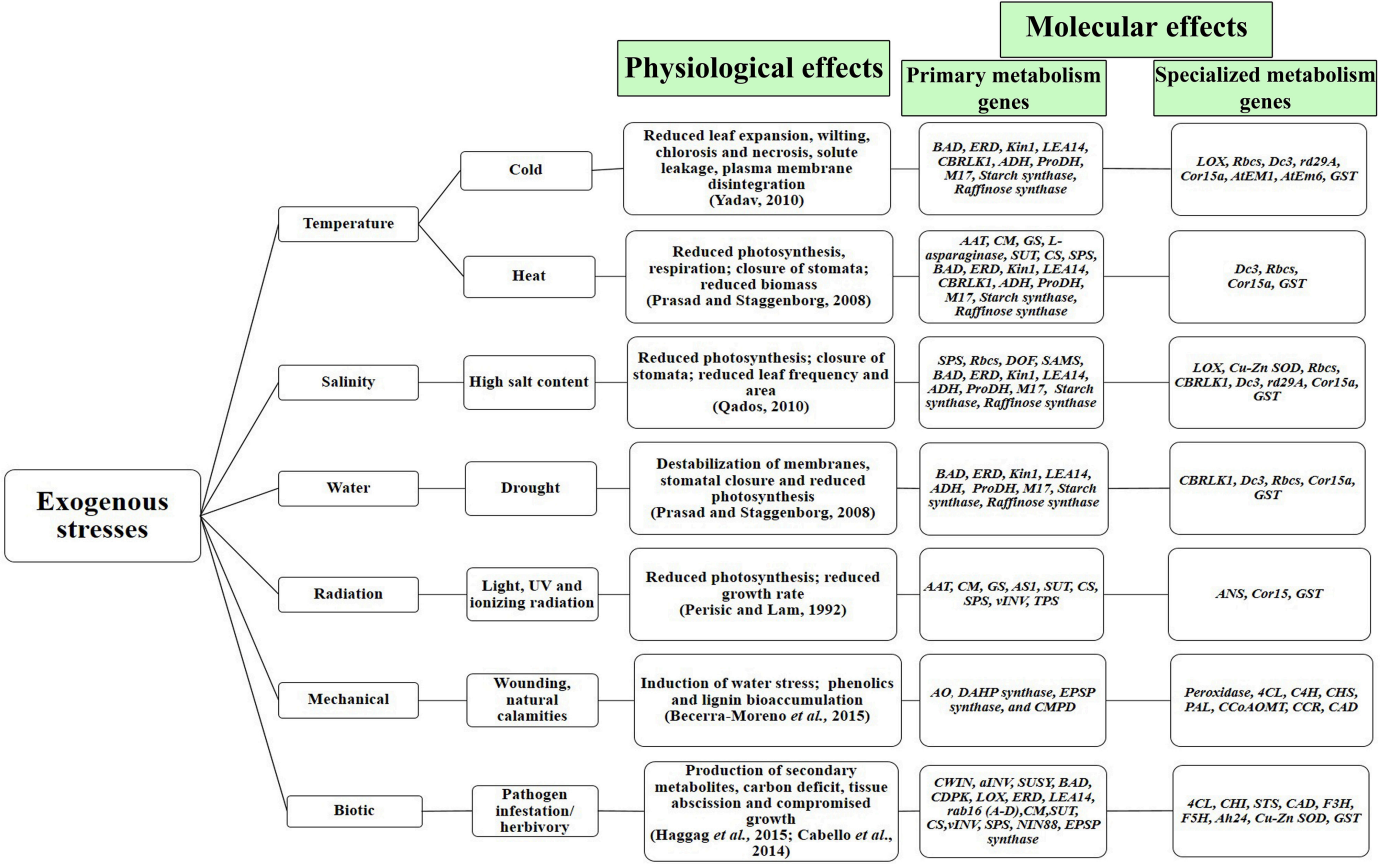
**Physiological and molecular effects of phyto-stress**. Temperature, salinity and drought stress have similar physiological and molecular footprints. The depicted primary and specialized metabolism genes show synchronized upregulation under abiotic and biotic stress (Full forms, the detailed list of stress-regulated genes and their references have been given in Table 1).

Plant stress has been one of the most widely studied areas of biological research, wherein scientific efforts are involved in studying its effects and devising techniques toward its mitigation. As a direct consequence of stress, plants undergo gross biochemical, physiological and molecular changes (as depicted in Figure 2). Due to variations in the metabolic profile of plants under stressed conditions (as described in Supplementary Table 1), the natural requirement of free energy toward maintenance of homeostasis and growth-associated processes get lowered, thereby causing growth-retardation and reduction in the overall plant productivity (Caretto et al., 2015). Plants inherently possess various systems to protect themselves from different forms of stress. This exercise is a combination of a complex array of regulations that occur at various levels, i.e., at whole plant, tissue, cellular, sub-cellular, genetic and molecular levels (Shulze et al., 2005; Prasad et al., 2008; Yadav, 2010; Qados, 2011; Ramakrishna and Ravishankar, 2011; Rejeb et al., 2014). Primarily, plants combat stress by redirecting the metabolic machinery to overproduce certain defense-associated primary and specialized metabolites (Caretto et al., 2015). As seen in Supplementary Table 1, distinct forms of stress display similar metabolite profiles, belonging to primary and specialized metabolism. The elevated levels of diverse metabolites under similar conditions of stress may arise due to coregulation of biochemical pathways at the molecular level. For example, literature evidence points toward an increased accumulation of at least 15 amino acids (belonging to the primary metabolism) together with volatile organic compounds (VOCs) under drought stress (Joshi and Jander, 2009; Fraire-Velázquez et al., 2011; Gill and Tuteja, 2011; Álvarez et al., 2012; Du and Wang, 2012; Hayat et al., 2012; Kendziorek et al., 2012; Griesser et al., 2015; Hudson, 2015; Niinemets, 2015; Weldegergis et al., 2015). Additionally, it was noted that abiotic stresses like temperature and salinity could regulate the levels of other primary (sugar alcohols and sugars) and specialized (phenylpropanoids, alkaloids, etc.) metabolites. (Flores and Galston, 1982; Smith, 1984; Cho et al., 1999; Streeter et al., 2001; Weise et al., 2006; Cuevas et al., 2008; Rosa et al., 2009; Gill and Tuteja, 2010; Hochberg et al., 2013, 2015; Zhao et al., 2013; Alam et al., 2014; Mouradov and Spangenberg, 2014; Zhang et al., 2014; Le Gall et al., 2015; Saleh and Madany, 2015; Sheshadri et al., 2015; Wei et al., 2015). Furthermore, biotic stress like herbivory also displayed a remarkably similar metabolite profile in plants; while primary metabolites like phenylalanine and allantoin were found to be elevated, the levels of VOCs (specialized metabolites) were also enhanced (Fraire-Velázquez et al., 2011; Du and Wang, 2012; Hayat et al., 2012; Kendziorek et al., 2012; Griesser et al., 2015; Hudson, 2015; Weldegergis et al., 2015; Takagi et al., 2016). Thus, the trend of overproduction of primary and specialized metabolites arising from diverse pathways under similar conditions of stress, further confirms the predominant role of stress as a possible link to elucidate the crosstalk between primary and specialized metabolism (Tuteja, 2007; Bolton, 2009; Qados, 2011; Bhargava and Sawant, 2013; Chamoli and Verma, 2014).

The process of stress tolerance in plants principally involves the regulation of stress-responsive genes that encode for primary metabolites, specialized metabolites and TFs (Davuluri et al., 2003; Floris et al., 2009; Osakabe et al., 2014). This advantage combined with various inherent signaling mechanisms (like pH, metal ions, symbionts, etc.) causes the upregulation of several cascade of genes in plant systems (Tuteja, 2007; Palmieri et al., 2008; Rushton et al., 2012). The mechanism adopted by these genes in bringing about stress tolerance depends on their inherent function, type of stress and the plant system (Davuluri et al., 2003; Shinozaki and Yamaguchi-Shinozaki, 2007; Floris et al., 2009; Osakabe et al., 2014). Functionally, majority of these genes are involved directly in stress mitigation by regulating physiological parameters like water homeostasis and osmoregulation *via* endogenous signaling (Tuteja, 2007). Table 1 illustrates the predominantly studied primary, specialized and TF genes that are coregulated under different stress conditions.

**Table 1:** **Predominant primary metabolism, specialized metabolism and TF genes coregulated under similar stress conditions**.

**Stress → Genes↓**	**Function**	**D**	**C**	**H**	**S**	**L**	**W**	**OA**	**Bio**	**References**
**PRIMARY METABOLISM GENES^#^**										
*Cell Wall Invertase (CWIN)*	Sucrose → D-Glucose + D-Fructose [KEGG reaction: R00801]	D	C	H	S	L	W	OA	Bio	Ciereszko et al., 2001; Proels and Roitsch, 2009; Hayes et al., 2010; Payyavula et al., 2013; Cabello et al., 2014; French et al., 2014; Chen et al., 2015; Niu et al., 2015
*Sucrose synthase (SUSY)*	UDP-D-Glucose + D-Fructose → Sucrose + UDP [KEGG reaction: R06036]	**D**	C	**H**	S	L	W	OA	Bio	Ahmadi and Baker, 2001; Ciereszko et al., 2001; Cabello et al., 2014; Le Gall et al., 2015; Peng et al., 2016
*Betaine aldehyde dehydrogenase (BAD)*	Betaine aldehyde + NAD^+^ + H_2_O → Betaine + NADH + 2 H^+^ [KEGG reaction: R02565]	D	C	H	S	L	W	OA	Bio	Gupta and Kaur, 2005; Zhang et al., 2008; Hasthanasombut et al., 2011; Stiti et al., 2011; Chen et al., 2014
*Late Embryogenesis Abundant (LEA14)*	Prevents protein aggregation under osmotic/cold stress	D	C	H	S	L	W	OA	Bio	Kimura et al., 2003; Pedrosa et al., 2015
*Aspartate kinase (AK)*	ATP + L-aspartate → ADP + 4-phospho-L-aspartate [KEGG reaction: R00480]	D	C	H	S	L	W	OA	Bio	Bastías et al., 2011, 2014; Yoshida et al., 2015
*Aspartate aminotransferase (AAT)*	L-aspartate + 2-oxoglutarate → oxaloacetate + L-glutamate [KEGG reaction: R00355]	D	C	H	S	L	W	OA	Bio	Bastías et al., 2011, 2014; Yoshida et al., 2015
*Chorismate mutase (CM)*	Chorismate → Prephenate (KEGG reaction: R01715)	D	C	H	S	L	W	OA	Bio	Bastías et al., 2011, 2014; Yoshida et al., 2015
*Glutamine synthetase (GS)*	ATP + L-glutamate + NH_3_ → ADP + phosphate + L-glutamine [KEGG reaction: R00253]	D	C	H	S	L	W	OA	Bio	Bastías et al., 2011, 2014; Yoshida et al., 2015
*Glutamine dehydrogenase (GDH)*	L-glutamate + H_2_O + NAD^+^ → 2-oxoglutarate + NH_3_ + NADH + H^+^ [KEGG reaction: R00243]	D	C	H	S	L	W	OA	Bio	Bastías et al., 2011, 2014; Yoshida et al., 2015
*Asparagine synthetase (AS1)*	ATP + L-aspartate + L-glutamine + H2O → AMP + diphosphate + L-asparagine + L-glutamate [KEGG reaction: R00578]	D	C	H	S	L	W	OA	Bio	Bastías et al., 2011, 2014; Yoshida et al., 2015
*Sucrose transporter (SUT)*	Facilitate active transport of sucrose across plasma membrane	D	C	H	S	L	W	OA	Bio	Bastías et al., 2011, 2014; Yoshida et al., 2015
*Citrate synthase (CS)*	ADP + phosphate + acetyl-CoA + oxaloacetate → ATP + citrate + CoA [KEGG reaction: R00352]	D	C	H	S	L	W	OA	Bio	Bastías et al., 2011, 2014; Yoshida et al., 2015
*Vacuolar invertase (vINV)*	Sucrose → D-Glucose + D-Fructose [KEGG reaction: R00801]	D	C	H	S	L	W	OA	Bio	Ciereszko et al., 2001; Proels and Roitsch, 2009; Hayes et al., 2010; Cabello et al., 2014; Rabot et al., 2014; Niu et al., 2015
*Starch Branching Enzyme (SBE)*	Amylose → Starch [KEGG reaction: R02110]	D	C	H	**S**	L	W	OA	Bio	Kim and Guiltinan, 1999; Theerawitaya et al., 2012
*Sucrose phosphate synthase (SPS)*	UDP-glucose + D-fructose 6-phosphate → UDP + sucrose 6'-phosphate [KEGG reaction: R00766; R06073]	D	C	H	S	L	W	OA	Bio	Roy Choudhury et al., 2008; Bastías et al., 2014; Morkunas and Ratajczak, 2014
*NIN88 (Tobacco invertase)*	Sucrose → D-Glucose + D-Fructose [KEGG reaction: R00801]	D	C	H	S	L	W	OA	Bio	Iven et al., 2010
*Alcohol dehydrogenase (ADH)*	Primary alcohol + NAD^+^ → an aldehyde + NADH + H^+^ [KEGG reaction: R00623]	D	C	H	S	L	W	OA	Bio	Lu et al., 1996; Kato-Noguchi, 2001; Sibéril et al., 2001; Jin et al., 2016
*Proline dehydrogenase (ProDH)*	L-proline + a quinone → (S)-1-pyrroline-5-carboxylate + a quinol [KEGG reaction: R01253]	D	C	H	S	L	W	OA	Bio	Satoh et al., 2004
*Ascorbate oxidase (AO)*	4 L-ascorbate + O_2_ → 4 monodehydroascorbate + 2H_2_O [KEGG reaction: R00068]	D	C	H	S	L	W	OA	Bio	Asao et al., 2003
*Dc3 (LEA class gene)*	Prevents protein aggregation under osmotic/cold stress	D	C	H	S	L	W	OA	Bio	Finkelstein and Lynch, 2000; Kim et al., 2002
*AtEM1(LEA class gene)*		D	C	H	S	L	W	OA	Bio	Finkelstein and Lynch, 2000
*M17 (LEA class gene)*		D	C	H	S	L	W	OA	Bio	Finkelstein and Lynch, 2000
*AtEm6 (LEA class gene)*		D	C	H	S	L	W	OA	Bio	Finkelstein and Lynch, 2000; Kim et al., 2002
*Starch synthase (ZmDULL1)*	ADP- α-D-glucose + [(1 → 4)-α-D-glucosyl]_n_ → ADP + [(1 → 4)- α-D-glucosyl]_n_ + 1 [KEGG reactions: R02421, R06049]	D	C	H	S	L	W	OA	Bio	Wu et al., 2015
α-Amylase (Amy3D)	Starch → Maltose + Dextrin [KEGG reaction: R02112]	D	C	H	S	L	W	OA	Bio	Hwang et al., 1998; Ashraf et al., 2002
*Raffinose synthase (ZmRS1, ZmRS2, ZmRS3 and ZmRS10)*	α-D-galactosyl-(1 → 3)-1D-myo-inositol + sucrose → myo-inositol + raffinose	D	C	H	S	L	W	OA	Bio	Zhou et al., 2012
*Trehalose phosphate synthase (TPS)*	UDP-glucose + D-glucose 6-phosphate → UDP + α, α-trehalose 6-phosphate [KEGG reactions: R00836, R06043]	D	C	H	S	L	W	OA	Bio	Henry et al., 2014
*Horseradish Peroxidase (HRP)*	2 phenolic donor + H_2_O_2_ → 2 phenoxyl radical of the donor + 2 H_2_O [KEGG reaction: R03532]	D	C	H	S	L	W	OA	Bio	Kawaoka et al., 1994; Caverzan et al., 2012
*DAHP synthase*	phosphoenolpyruvate + D-erythrose 4-phosphate + H_2_O → 3-deoxy-D-arabino-hept-2-ulosonate 7-phosphate + phosphate [KEGG reaction: R01826]	D	C	H	S	L	W	OA	Bio	Schlüter et al., 2013; Becerra-Moreno et al., 2015
*EPSP synthase*	phosphoenolpyruvate + 3-phosphoshikimate → phosphate + 5-O-(1-carboxyvinyl)-3-phosphoshikimate [KEGG reaction: R03460]	D	C	H	S	L	W	OA	Bio	Becerra-Moreno et al., 2015
*Chorismate mutase prephenate dehydratase (CMPD)*	Chorismate → Prephenate Prephenate → Phenylpyruvate	D	C	H	S	L	W	OA	Bio	Becerra-Moreno et al., 2015
**SPECIALIZED METABOLISM GENES^#^**										
*4-coumarate coenzyme A ligase (4CL)*	ATP + 4-coumarate + CoA → AMP + diphosphate + 4-coumaroyl-CoA [KEGG reaction:R01616]	D	C	H	S	L	W	OA	Bio	Neustaedter et al., 1999; Soltani et al., 2006; Chowdhury et al., 2012; Kim et al., 2013; Le Gall et al., 2015
*Chalcone isomerase (CHI)*	a chalcone → a flavanone [KEGG reaction: R07344]	D	C	H	S	L	W	OA	Bio	Ahn et al., 2014
*Stilbene synthase (STS)*	3 malonyl-CoA + cinnamoyl-CoA → 4 CoA + pinosylvin + 4 CO_2_ [KEGG reaction: R02505] 3 malonyl-CoA + 4-coumaroyl-CoA → 4 CoA + trans-resveratrol + 4 CO_2_ [KEGG reaction: R01614]	D	C	H	S	L	W	OA	Bio	Ahn et al., 2014
*Caffeoyl-CoA O-methyltransferase (CCoAOMT)*	S-Adenosyl-L-methionine + Caffeoyl-CoA ↔ S-Adenosyl-L-homocysteine + Feruloyl-CoA	D	C	H	S	L	W	OA	Bio	Chowdhury et al., 2012; Le Gall et al., 2015
*Cinnamyl alcohol dehydrogenase (CAD)*	cinnamyl alcohol + NADP^+^ → cinnamaldehyde + NADPH + H^+^ [KEGG reaction: R03054]	D	C	H	S	L	W	OA	Bio	Kim et al., 2013
*Cinnamate-4-monooxygenase (C4H)*	trans-cinnamate + NADPH + H^+^ + O_2_ → 4-hydroxycinnamate + NADP+ + H_2_O [KEGG reaction: R02253]	D	C	H	S	L	W	OA	Bio	Kim et al., 2013; Becerra-Moreno et al., 2015; Le Gall et al., 2015
*Dihydroflavonol 4-reductase (DFR)*	a (2R,3S,4S)-leucoanthocyanidin + NADP^+^ → a (2R,3R)-dihydroflavonol + NADPH + H^+^ [KEGG reaction: R03123]	D	C	H	S	L	W	OA	Bio	Tsai et al., 2006; Singh et al., 2009; Payyavula et al., 2013; Ahmed et al., 2014
*Chalcone synthase (CHS)*	3 malonyl-CoA + 4-coumaroyl-CoA → 4 CoA + naringenin chalcone + 3 CO_2_ [KEGG reaction: R01613]	D	C	H	S	L	W	OA	Bio	Lawton and Lamb, 1987; Ahn et al., 2014
*Flavanone 3-hydroxylase (F3H)*	a flavanone + 2-oxoglutarate + O_2_ → a dihydroflavonol + succinate + CO_2_ [KEGG reaction: R07329]	D	C	H	S	L	W	OA	Bio	Xie et al., 2012; Payyavula et al., 2013
*Ferulate 5-hydroxylase (F5H)*	Catalyzes rate-limiting step in syringyl lignin biosynthesis pathway; required for production of sinapate esters	D	C	H	S	L	W	OA	Bio	Chowdhury et al., 2012; Le Gall et al., 2015
*Hydroxy cinnamoyl transferase (HCT)*	4-coumaroyl-CoA → 4-coumaroyl-shikimate/quinate	D	C	H	S	L	W	OA	Bio	Chowdhury et al., 2012; Kim et al., 2013; Payyavula et al., 2013
*Coumarate 3-hydroxylase (C3H)*	4-coumaroyl-shikimate/quinate → caffeoyl-shikimate/quinate	D	C	H	S	L	W	OA	Bio	Chowdhury et al., 2012
*12-oxophytodienoate (OPR)*	8-[(1R,2R)-3-Oxo-2-{(Z)-pent-2-enyl}cyclopentyl]octanoate + NADP^+^ ↔ (15Z)-12-oxophyto-10,15-dienoate + NADPH + H^+^ [KEGG reaction: R03401]	D	C	H	S	L	W	OA	Bio	Diaz et al., 2012
*Phenylalanine Ammonia Lyase (PAL)*	L-phenylalanine → trans-cinnamate + NH_3_ [KEGG reaction: R00697]	D	C	H	S	L	W	OA	Bio	Lawton and Lamb, 1987; Chowdhury et al., 2012; Kim et al., 2013; Payyavula et al., 2013
*Lipoxygenase (LOX)*	Linoleate + O_2_ → (9Z,11E,13S)-13-hydroperoxyoctadeca-9,11-dienoate [KEGG reaction: R03626]	D	C	H	S	L	W	OA	Bio	Nemchenko et al., 2006; Umate, 2011; Padilla et al., 2014
*Amaranthus hypochondriacus unknown protein (Ah24)*	Stress-responsive protein	D	C	H	S	L	W	OA	Bio	Massange-Sanchez et al., 2015
*Anthocyanidin synthase (ANS)*	Leucocyanidin + 2-oxoglutarate + O_2_ → cis- and trans-dihydroquercetins + succinate + CO_2_ + 2H_2_O [KEGG reactions: R05723, R07366]	D	C	H	S	L	W	OA	Bio	Mellway et al., 2009
*Cu-Zn superoxide dismutase (Cu-Zn SoD)*	2 superoxide + 2 H^+^ → O_2_ + H_2_O_2_ [KEGG reaction: R00275]	D	C	H	S	L	W	OA	Bio	Qu et al., 2010
*Glutathione-S-transferase (GST)*	R-X+Glutathione ↔ Halide + R-S- Glutathione [R = side chain; X = halogen; KEGG reaction: R03522]	D	C	H	S	L	W	OA	Bio	Marrs, 1996; Uquillas et al., 2004; Gupta and Kaur, 2005
*S-adenosyl methionine decarboxylase (SamDC)*	S-adenosyl-L-methionine → S-adenosyl 3-(methylthio)propylamine + CO_2_ [KEGG reaction: R00178]	D	C	H	S	L	W	OA	Bio	Yoshida et al., 1998; Li and Chen, 2000; Rodriguez-Kessler et al., 2006; Bae et al., 2008; Chamoli and Verma, 2014
*S-Adenosyl-L-methionine synthase (SAMS)*	ATP + L-methionine + H_2_O → phosphate + diphosphate + S-adenosyl-L-methionine [KEGG reaction: R00177]	D	C	H	S	L	W	OA	Bio	Sánchez-Aguayo et al., 2004; Kim et al., 2013
**TRANSCRIPTION FACTOR GENES^#^**										
*Methyl Jasmonate induced MYB-related TF (MYBJS)*	Circadian clock regulation	D	C	H	S	L	W	OA	Bio	Gális et al., 2006; Zhao and Dixon, 2011; Höll et al., 2013; Payyavula et al., 2013
*Basic loop helix (StBHLH)*	Cell activity and developmental regulation, circadian clock	D	C	H	S	L	W	OA	Bio	Payyavula et al., 2013; Babitha et al., 2015; Sun H. et al., 2015
*WRKY*	Regulation of stress response, seed development and senescence control	D	C	H	S	L	W	OA	Bio	Teixeira et al., 2014; Banerjee and Roychoudhury, 2015
*Anthocyanin1 (StAN1)*	Activates transcription of structural anthocyanin genes	D	C	H	S	L	W	OA	Bio	Payyavula et al., 2013
*WD40*	Regulation of cell division, vesicle formation, signal transduction and processing of RNA	D	C	H	S	L	W	OA	Bio	Payyavula et al., 2013
*DOF*	Regulation of light and phytohormone response, seed maturation and germination	D	C	H	S	L	W	OA	Bio	Noguero et al., 2013; Ma et al., 2015
*bZIP*	Regulates pathogen defense, light and stress signaling, flower development and seed maturation	D	C	H	S	L	W	OA	Bio	Jakoby et al., 2002; Wei et al., 2012; Liu et al., 2014
**OTHER GENES^#^**										
*Responsive to Abscisic acid [rab-16 (A-D)]*	Regulation of stress tolerance and ABA response	D	C	H	S	L	W	OA	Bio	Mundy et al., 1990; Ganguly et al., 2012; Rabot et al., 2014
*Responsive to Drought (rd29A)*	ABA-responsive drought and desiccation tolerance	D	C	H	S	L	W	OA	Bio	Taji et al., 1999; Kimura et al., 2003; Das et al., 2014
*Cold responsive (COR15a)*	Cold and osmotic stress tolerance, red or far red light signaling pathway	D	C	H	S	L	W	OA	Bio	Kimura et al., 2003
*Calcium-dependent Protein Kinase (CPKI)*	Regulation of plant stress tolerance	D	C	H	S	L	W	OA	Bio	Campos-Soriano et al., 2011
*Kin1 (stress-induced protein)*	Regulation of plant stress tolerance	D	C	H	S	L	W	OA	Bio	Wang et al., 1995; Kimura et al., 2003
*S-locus receptor-like protein kinase (CBRLK1)*	Negative regulator of disease resistance pathway in plants	D	C	H	S	L	W	OA	Bio	Das et al., 2014
*Ca^2+^-dependent, calmodulin independent protein kinase (CDPK)*	Regulation of light stress tolerance, seed development	D	C	H	S	L	W	OA	OA Bio	Frattini et al., 1999; Gupta and Kaur, 2005; Cai et al., 2015
*Early Responsive to Dehydration (ERD)*	Negative regulator of ABA response (resistance to drought, freezing and regulation of stomatal closure)	D	C	H	S	L	W	OA	Bio	Taji et al., 1999; Kimura et al., 2003

# *classification of genes into primary, specialized and TF clusters is based on literature evidence, KEGG map01100 and their function. D, drought; C, cold; H, heat; S, salinity; Bio, biotic stress; L, light; W, wounding; OA, other abiotic (like elicitors: ABA, jasmonic acid, salicylic acid, ethylene; exogenous chemical treatment like glucose/sucrose supplementation etc.); green box, upregulation; red box, downregulation; black box, differential/inconsistent expression; white box, information insufficient*.

The regulation of a wide spectrum of genes under stress occurs principally at the transcriptional level (Shinozaki and Yamaguchi-Shinozaki, 2007). This is especially brought about by TF binding to their specific *cis*-elements present in the 5′ flanking regions of gene(s) (Passricha et al., 2016). Moreover, the patterns of *cis*-elements present among the promoter and intronic regions decide the levels of gene expression (Rombauts et al., 2003; Brown et al., 2007; Zou et al., 2011; Hernandez-Garcia and Finer, 2014), and any mutation(s) occurring in this region can greatly influence the stress-responsiveness of the coded genes (Wittkopp and Kalay, 2012). Most notably, diverse forms of stress may activate similar *cis*-element TF regulatory networks (Faktor et al., 1996; Kim et al., 2006; Soltani et al., 2006; Mellway et al., 2009; Cao et al., 2012; Payyavula et al., 2013; Ahn et al., 2014; Chen et al., 2015; Zhu et al., 2015). Despite having a great depth of understanding on plant stress and its significance in connecting diverse pathways, not many reports are available that connect the primary and specialized metabolism at the transcriptional level. The forthcoming sections present an in-depth understanding of the molecular level regulation of stress-responsive genes, with an insight into the transcriptional regulation mediated by *cis*-element and TF interactions.

As observed from Table 1, the expression profile of genes show remarkable similarity among primary and specialized metabolism. Under similar stressed conditions, it could be observed that most of the primary, specialized and TF/other genes show enhanced expression. Literature evidences indicate that coexpression of wide spectrum of genes is a resultant of coregulation at the transcriptional level, primarily *via cis*-elements and TF interactions (Brown et al., 2007; Shinozaki and Yamaguchi-Shinozaki, 2007; Floris et al., 2009; Nakashima et al., 2009; Lata et al., 2011; Zou et al., 2011; Basu et al., 2014). The study involving *cis*-elements has gained impetus in the recent years, especially in elucidating the link between pathways which are known to be dependent on each other, but whose genetic inter-dependency is not much known. Bioinformatics has enabled researchers to elucidate and forecast the type of stress-responsive transcriptional regulation of genes by studying the pattern of *cis*-elements present in the upstream regions of these genes (Ibraheem et al., 2010). Several tools have been made available for this purpose, like Plant *Cis*-Acting Regulatory DNA Elements Database (http://www.dna.affrc.go.jp/PLACE; Lescot et al., 2002), Genomatix (http://www.genomatix.de; Cartharius et al., 2005) and *Arabidopsis* Gene Regulatory Information Server (AGRIS; http://arabidopsis.med.ohio-state.edu/; Davuluri et al., 2003). Based on a highly efficient Hidden Markov Model, a database of probable TF binding sites in the promoters of stress–responsive genes of *A. thaliana* is also available for further research (Malhotra and Sowdhamini, 2014). *Cis*-elements are further involved in imparting several auxiliary functions to the plant systems, like developmental regulation of growth associated processes, morphological modifications, regulating senescence, DNA damage repair mechanisms, etc. (Floris et al., 2009; Nakashima et al., 2009; Zou et al., 2011).

Among the stress-responsive genes enlisted in Table 1, the promoter analysis data was available for 33 genes, and their characteristic *cis*-elements have been shown in Supplementary Table 2. It can be inferred that there are several *cis*-elements that are commonly overrepresented in the promoter regions of various primary and specialized metabolism genes. Such elements can present a plausible molecular link between diverse pathways. As discussed earlier, these elements possess additional roles (like developmental regulation, controlling circadian cycle, etc.) apart from their characteristic role of stress-responsive transcriptional regulation. The promoter regions of most of the primary and specialized metabolism genes possessed following *cis*-elements: *ABRE, G-box, W-box* and *MYB-recognizing elements. ABRE* and *G-box* elements are favorable binding sites of bZIP TFs that regulate stress responses (Heinekamp et al., 2002, 2004). Studies have shown that one of the bZIP TFs, BZI-1 is involved in imparting auxin responsiveness and regulating pollen development *via* carbohydrate allocation (Heinekamp et al., 2004; Iven et al., 2010). BZI-1 TFs bind specifically to the *ACEs* (ACGT core elements; example: *G-box, GT-box*, etc.), thereby controlling stress-specific regulation of primary (NIN88, Adh, α-amylase, AtEM6, ProDH, Dc3, LEA, Kin1, BAD, GST and SbeI; Lu et al., 1996; Finkelstein and Lynch, 2000; Kim et al., 2002; Satoh et al., 2004; Wobbes, 2004; Iven et al., 2010; Bastías et al., 2011, 2014) and specialized metabolism genes (CHI, CHS, Ah24, DFR and PAL; Strathmann et al., 2001; Heinekamp et al., 2002, 2004; Fujita et al., 2005; Iven et al., 2010; Yoshida et al., 2015). It can therefore be inferred that the *ACEs* and bZIP TFs interactions can play a central role in coregulating primary and specialized metabolism in plants. In addition, the WRKY binding sites (*W-box*) were also found to be present in the promoter regions of some primary (*GST, ANS, SUSY, vINV* and *CWIN*) and specialized metabolism genes (*HCT, CHS, C3H, F3H, PAL* and *DFR*). Physiologically, the WRKY TFs binding to *W-box*es regulate various developmental activities (trichome development and controlling senescence) and defense associated processes (like regulating responses to pathogen infestation and other abiotic stresses) (Aken et al., 2013; Llorca et al., 2014). This mechanism of coregulating diverse genes under stressed conditions indicates at WRKY-*W-box* interactions as a prospective link between primary and specialized metabolism. Similarly, the MYB-binding sites are present in the promoters of several primary (*LEA14, CWIN, vInv 1* and *SUSY*) and specialized metabolism genes (*CHI, HCT, ANS, DFR, F3H, PAL, C3H* and *GST*). Moreover, it is known that MYB TFs binding to their respective *cis*-elements regulate changes in various processes like hormonal signaling, specialized metabolism (phenylpropanoid and anthocyanin biosynthesis), cellular morphogenesis, and formation of meristem (Cao et al., 2013; Höll et al., 2013). This striking similarity observed in the promoter regions of functionally distinct genes provides ample scope to draw a link between diverse pathways at the transcriptional level *via cis*-element-TF interactions serving as the bridges.

### TF families regulating stress-mediated link between primary and specialized metabolism

In plants, the transcriptional mode of gene regulation is mediated synergistically by a combination of TFs acting in tandem to bring about different expression patterns. Studies have revealed that in *Arabidopsis*, about 5–10% of the functional genes are TFs, which regulate diverse genes under different environmental conditions (Mitsuda and Ohme-Takagi, 2009). The most studied stress-responsive TFs principally belong to six families, namely bZIP, WRKY, MYB, APETALA2 (AP2 family), NAC and Zinc finger family (ZnF) (Saibo et al., 2009; Gujjar et al., 2014; Malhotra and Sowdhamini, 2014). However, the largest TF families- bZIP, WRKY, MYB and AP2 are more extensively involved in regulating diverse metabolic pathways in plants under stress (Heinekamp et al., 2002; Jakoby et al., 2002; Katiyar et al., 2012; Rushton et al., 2012; Wei et al., 2012; Alves et al., 2013; Llorca et al., 2014; Liu et al., 2015; Wang et al., 2015). Also, most TFs can recognize secondary motifs (apart from their primary recognition sequences), which allow them to bind to distinct sites in the promoters. Further, TFs having upto 79% amino acid similarity in their recognition domain have shown distinct DNA binding profiles. Several other TFs like ERFs, bZIPs, etc. also demonstrated their ability to recognize and bind to secondary motifs which partially differ from their respective primary motifs (Franco-Zorrilla et al., 2014). The forthcoming sections describe the mode of action of the above four predominant TF family proteins and their role in simultaneously regulating primary and specialized metabolism genes.

#### The bZIP family

The bZIP family (basic leucine zipper) is one of the largest TF families in plants, which is involved in diverse regulatory functions, like abiotic and biotic stress tolerance, hormone signaled gene regulation, sugar signaling, nitrogen, carbon and energy metabolism, light responsiveness and developmental regulation (like cell elongation, differentiation, flowering, senescence and maturation of seedlings, Chuang et al., 1999; Wei et al., 2012; Bastías et al., 2014; Llorca et al., 2014; Zhao et al., 2016). The bZIP TFs have a widespread presence among eukaryotes (17 in *S. cerevisiae*, 27 in Drosophila, 75 in *A. thaliana*, 89 in rice, 125 in maize, 131 in soybean, 69 in tomato and 585 among six leguminous plants: *G. max, M. truncatula, P. vulgaris, C. arietinum, C. cajan*, and *L. japonicus*, Fassler et al., 2002; Wei et al., 2012; Llorca et al., 2014; Li D. et al., 2015; Wang et al., 2015). These TFs possess a binding affinity toward the core motif–ACGT- (*ACEs*), which is found in *G-Box, A-Box, C-Box* and *ABRE*.

bZIP TFs are comprised of a short basic region linked to a DNA recognition domain followed by a leucine repeat region that imparts amphipathic nature to the protein (Jakoby et al., 2002; Alves et al., 2013; Llorca et al., 2014). The leucine zipper region of the protein binds to the bZIP recognition sequences in a chopstick fashion (Sibéril et al., 2001; Iven et al., 2010; Alves et al., 2013; Llorca et al., 2014). bZIP TFs can be sub-classified into 10 groups, out of which groups A, C, D, G and S have been studied extensively (Jakoby et al., 2002; Dey et al., 2016). Table 2 describes these groups with special emphasis on its relevance in simultaneously regulating primary and specialized metabolism genes.

**Table 2:** **bZIP TF family in regulating primary and specialized metabolism genes simultaneously**.

**S. N**.	**bZIP TF**	**Functional homologs**	**Recognition sequence (5′—3″)**	**Primary metabolism genes**	**Specialized metabolism genes**	**References**
**GROUP-A bZIPs**						
1	AREB (ABRE-binding proteins)	AREB1/ABF2, AREB2/ABF4, ABF3	CACGTGGC	*SUSY, LEAs, CWIN, vINV, rbcS, PP2C, OsRab16B, OsRab21*	*PAL, CHS, DFR, FLS*	Perisic and Lam, 1992; Tsai et al., 2006; Hundertmark and Hincha, 2008; Bastías et al., 2014; Zhang et al., 2014; Dey et al., 2016
**GROUP-C bZIPs**						
2	Opaque2 (O2)	AtbZIP10, AtbZIP25	TCCACGTAGA	*Tryp synthase, SusI, Adh, α-zein Z1, b32 albumin, malate dehydrogenase, α-galactosidase, Starch synthase, SPS, Citrate synthase, Xylose isomerase*	*DFR, CS1, IDI-1, PAL*	Schmidt et al., 1992; Hunter et al., 2002; Jakoby et al., 2002; Bhat et al., 2004; Hartings et al., 2011
**GROUP-D bZIPs**						
3	PERIANTHIA (AtbZIP46)	TGA1, HBP-1b, OBF 3.1, OBR 3.2	TGACGT*/*C	*AG, STP4, PSD1, FSD1*	*IFR, PR-1*	Maier et al., 2009, 2011
**GROUP-G bZIPs**						
4	GBF (*G-Box* binding factor)	GBF1, GBF2, GBF3, GBF4	CACGTG	*Em genes, GH3, Adh, SBE*	*CHS, CHI, PAL, DFR, ANS*	Lu et al., 1996; Sibéril et al., 2001; Heinekamp et al., 2004
5	BZI-1, BZI-2	G/HBF-1, CPRF2, TBZF	G/CACGTG	*GH3, NIN88, AtcwINV2*	*CHS, PAL*	Heinekamp et al., 2004; Iven et al., 2010
**GROUP-S bZIPs**						
6	ATB2	AtbZIP11	TGACGTG; ACTCAT	*ProDH, CWIN, SUT, AS1*	Not available	Satoh et al., 2004; Wobbes, 2004; Hanson et al., 2008

*AG, Agamous gene; FSD1, Fe superoxide dismutase 1; GH3, Gretchen Hagen3; IDI-1, Isopentenyl diphosphate isomerase I; IFR, Isoflavone reductase; PP2C, group A type 2C phosphatase; PSD1, Phosphatidylserine decarboxylase; STP4, Sucrose transporter 4; SPS, Sucrose phosphate synthase; Tryp, Tryptophan*.

Among the bZIPs, varied *cis*-element and TF binding patterns bring about differential expression of diverse stress-responsive genes. For example, studies have indicated that the expression of primary (*SUSY, LEAs, CWIN, vINV, PP2C*) and specialized metabolism genes (*PAL, CHS, DFR, FLS*) is regulated by AREB-*ABRE* interactions *via* ABA signaling (Narusaka et al., 2003; Gómes-Porras et al., 2007; Bastías et al., 2011, 2014; Basu et al., 2014; Yoshida et al., 2015). Similarly, Opaque 2 (O2) is an endosperm-specific TF that was found to enhance the expression of several primary and specialized metabolism genes (Table 2). O2 TF binding to its recognition sites is mediated by certain transcriptional coactivators (like GCN5 and ADA2), which acetylate histone residues and thereby reinforce the binding. Further, a loss-of-function *O2* mutant severely impaired several developmental activities (like seed storage by downregulating storage protein coding genes; Schmidt et al., 1992; Schmitz et al., 1997; Zhang et al., 2015) and defense processes (Hunter et al., 2002; Bhat et al., 2004; Hartings et al., 2011). Further, one of the group-D bZIP TFs, PERIANTHIA was found to regulate developmental processes like controlling floral organ number (*via* regulation of the MADS domain TF gene *Agamous;* Maier et al., 2009), shoot meristem regulation (*via* FEA4, an ortholog of PERIANTHIA, Pautler et al., 2015) and regulating pathogen defense responses (Maier et al., 2011). It is associated with TGA regulators which is known to act upstream to the *PR* (Pathogenesis-related) gene, thereby conferring pathogen responsiveness to the plant systems. It was further observed that PERIANTHIA TF binds to the promoter regions of several primary and specialized metabolism genes, thereby causing their simultaneous regulation (Table 2). This pattern of simultaneous regulation of primary and specialized metabolism genes is demonstrated by several other bZIP TFs as well (GBFs, BZI, etc.; Lu et al., 1996; Sibéril et al., 2001; Heinekamp et al., 2002, 2004; Iven et al., 2010). HY5, a bZIP TF known to induce chlorophyll and carotenoid genes in plants, acts as a bridge between ABA and GA signaling pathways (owing to the fact that GA and ABA share the common precursor, Geranyl geranyl diphosphate; Mohanty et al., 2016). Despite being associated largely with phytohormone mediated stress responses, the bZIP family is involved in regulating growth and developmental activities like flowering, senescence, seed storage regulation, etc. (Rook et al., 1998; Hunter et al., 2002; Hanson et al., 2008; Hartings et al., 2011). The similarity in the pattern of occurrence of bZIP recognition sites (*ACEs*) among the promoters of primary and specialized metabolism genes depict bZIP-*cis*-elements interactions as a credible link to bridge diverse metabolic pathways *in planta*.

Although several reports indicated positive regulation of downstream genes by bZIP TFs, it has been observed that overexpressing BZI-4 (a bZIP TF that possesses strong affinity toward *G-Box* element) caused significant reduction in the expression of *NIN88* (Iven et al., 2010). Evidence suggests that BZI subfamily possesses conflicting roles in regulating developmental processes. Although BZI-1 and BZI-2 are involved in the transcriptional activation of *NIN88* gene, homo-dimerized BZI-4 acts as a repressor (Iven et al., 2010). Furthermore, despite the S-group being the largest bZIP subgroup in *Arabidopsis* (Jakoby et al., 2002), its significance in regulating specialized metabolism processes have not been studied much. Therefore, an in-depth research into this area needs to be conducted to understand the finer details on bZIP TFs and their involvement in linking the primary and specialized metabolism *in planta*.

#### The WRKY family

WRKY protein family in model plants *A. thaliana* and *N. benthamiana* is one of the largest TF families, which majorly bind to the *W-box*, a 6-bp region (C/TTGACC/T) present in the promoters of various primary and specialized metabolism genes. This has been known to bring about tolerance to abiotic and biotic stresses and regulate developmental processes in plants (trichome development and senescence; Basu et al., 2014; Llorca et al., 2014). *W-boxes* are present in the upstream regions of several genes like *PR1, isochorismate synthase 1* and ABA responsive genes: *SamDC, RD29A, COR47, iso1*, etc. (Supplementary Table 2; Sun et al., 2003; Rushton et al., 2012; Aken et al., 2013; Basu et al., 2014; Llorca et al., 2014; Singh and Laxmi, 2015). Although every WRKY TF has an affinity toward *W-Box*, they also possess additional DNA sequence affinities (like SUSIBA2 and SURE, sucrose responsive elements, core motif TGGACGG; Sun et al., 2003; Bi et al., 2016). The nucleotide sequences flanking the core *W-Box* element also decide the binding specificity of WRKYs. Most WRKYs are induced by plant hormones, like SA, ABA, etc. However, research reports highlight that SA induction and subsequent binding is more evident for extended *W-box*es (Franco-Zorrilla et al., 2014). Since majority of WRKYs bind to *W-Box*es to bring about transcriptional and posttranscriptional regulation of diverse genes, plants have developed an extrinsic mechanism to eliminate non-specific binding of repressor WRKYs to cause the activator WRKY to fit in and perform the stress-responsive gene regulation (Llorca et al., 2014).

**Figure 3:**
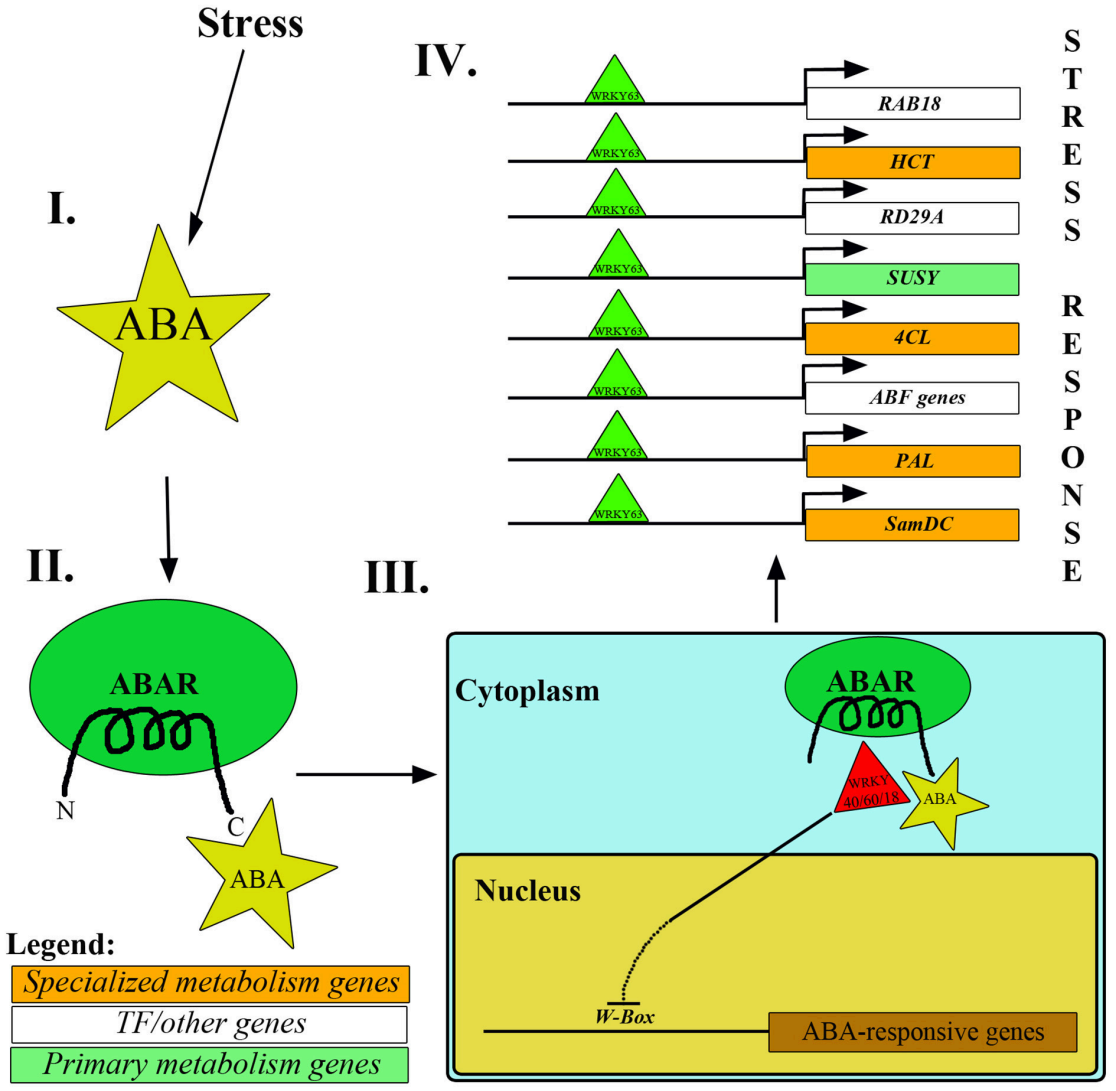
**Mechanism of repressor WRKYs removal from nucleus mediated by ABA. (I, II)**. Under stress conditions, ABA binds to the C-terminus of ABAR. **(III)** Consequently, it leads to the transport of *At*WRKY40/18/60 from the nucleus to the cytoplasm. **(IV)** Subsequently, *At*WRKY63 binds to the promoter regions of stress-responsive genes like *RAB18, RD29A, HCT, SUSY, 4CL, PAL, SamDC* and *ABF* genes, thereby enhancing their expression and mitigating stress.

One of the mechanisms involved in upregulation of stress-responsive genes is *via* ABA, which triggers the removal of repressor WRKYs from the promoter regions of ABA responsive genes (*ABF4, ABI4, DREB1a, MYB2, RAB18*; Rushton et al., 2012; Aken et al., 2013). According to studies, some WRKY proteins (*At*WRKY40, *At*WRKY18 and *At*WRKY60) disallow the transcription of ABA responsive genes upon binding to the *W-box* sequence [(C/T)TGAC(T/C)] in the promoter region. To ensure successful ABA-mediated stress mitigation, these WRKY TFs are translocated from the nucleus to the cytosol by making use of the affinity between the C-terminus of ABA-bound ABA receptor and WRKY proteins (Rushton et al., 2012; Aken et al., 2013). ABAR (or Mg-chelatase H-subunit/putative ABA receptor) is a chloroplast membrane-localized receptor which exposes its N and C-termini to the cytoplasm. ABA, upon binding to the C-terminal of ABAR, promotes the exit of WRKY proteins (WRKY40, WRKY18 and WRKY60) from the nucleus to cytosol, thereby facilitating the enhanced expression of ABA-responsive genes *via* binding of other activator WRKY TFs (example, WRKY63) to the *W-box* in the promoter regions (Figure 3; Rushton et al., 2012; Aken et al., 2013).

ABA-mediated WRKY-*W box* binding can form a crucial link between primary/growth-associated metabolic processes and stress-responsive/defense pathways. ABA helps mitigate drought through closure of guard cells of the stomata (Tuteja, 2007; Yoshida et al., 2015), simultaneously regulating the expression of several drought associated and cold stress responsive genes (*ABI genes, MYB2, RAB18, RD29A, ABF4, AOX1, DREB2*, etc.; Rushton et al., 2012; Qin et al., 2015). The occurrence of *ABRE* and *W-box* elements upstream to the coding regions of specialized metabolism genes further determines the plant response to ABA under stress (Fujita et al., 2005; Gómes-Porras et al., 2007; Yoshida et al., 2015). Additionally, reports suggest that in *O. sativa*, the presence of *W-box* elements upstream to the polyamine synthesis gene (*SamDC*) plays a key role in its upregulation (Basu et al., 2014). Furthermore, *Ta*WRKY93 (WRKY protein from wheat) was found to enhance the levels of Pyrroline-5-carboxylate synthase (P5CS; Qin et al., 2015) involved in proline biosynthesis (Proline is known to be directly involved in drought mitigation as osmoticum; Chamoli and Verma, 2014). It was also noted that WRKYs have a significant role in upregulating several other stress-responsive genes (like *ABF3, ABIs, DREB2A, RDs*, etc.; Qin et al., 2015). The synergistic binding of WRKY to *W-box*; ABF to *ABRE*; MYB TFs to *MYB* recognition elements; CBFs to *LTRE* and GBFs to *GATA* was found to upregulate *SamDC* gene in *O. sativa* (Basu et al., 2014). Under *P. infestans* infection, *St*WRKY1 was found to regulate the levels of *4CL* and *HCT* by binding to the *W-box*es present in their promoters (Yogendra et al., 2015).

Scientific evidence suggests that WRKY operation is often synergistically linked to the occurrence of ABA responsive bZIP TFs (Llorca et al., 2014). The pattern of occurrence of similar *cis*-elements among the upstream sequences of genes constituting diverse pathways (primary metabolism: *Invertases, SUSY, SUT*; specialized metabolism: *DREB1a, PAL, SamDC*; Yogendra et al., 2015) leads to a substantial hypothesis that these TF-*cis*-element interactions can form a regulatory bridge between primary and specialized metabolism. However, the detailed mechanism of WRKY as a plausible link between primary and specialized metabolism genes yet needs to be unraveled.

#### The MYB family

The MYB TF family is also one of the largest TF families in plants. As many as 125 in *A. thaliana* (Stracke et al., 2007), 205 in *G. raimondii* (He et al., 2016) and 559 in *S. lycopersicum* (Gates et al., 2016). Based on the number of MYB domains they contain, MYB TF family can be subdivided into four sub-families, namely 1R (R1/2/3), 2R (R2R3), 3R (R1R2R3) and 4R (R1R2R2R1/2), among which the R2R3-MYBs form the largest population (56.77% in *O. sativa*; 70.05% in *A. thaliana*). Literature evidence strongly suggests the involvement of 2R (R2R3-MYBs) in regulating several diverse metabolic processes.

This subfamily of MYB proteins is known to bind to the *MYB-recognizing elements* (*MREs* having the consensus sequence ANCNNCC, as demonstrated in MBSI, MBSII and MBSIIG; Franco-Zorrilla et al., 2014; Zhu et al., 2015), regulate Phenylpropanoid metabolism in plants (Liu et al., 2015). Additionally, R2R3-MYBs have been associated with several pleiotropic roles, like cell wall synthesis, regulation of pollen wall composition, glucosinolate biosynthesis, developmental processes, responses to physiological stress and determination of cell fate and identity (Lu et al., 2002; Du et al., 2012; Cao et al., 2013; Höll et al., 2013; He et al., 2016; Gates et al., 2016).

The R2R3-MYB TFs that play a crucial role in transcriptional regulation of primary and specialized metabolism have been enlisted in Table 3. From the table, it can be inferred that although *At*MYB32, *At*MYB3, *At*MYB4, *At*MYB26/MS35, *At*MYB28, *At*MYB29, *At*MYB76, *At*MYB103, *At*MYB34, *At*MYB51 and *At*MYB122 have been associated largely with primary and developmental processes; *At*MYB58, *At*MYB63, *At*MYB75, *At*MYB85, *At*MYB68, *At*MYB111, *At*MYB114 and *At*MYB123 are involved much into regulating Phenylpropanoid pathway (lignin/anthocyanin biosynthesis processes).

**Table 3:** **R2R3-MYB TFs in regulating primary and specialized metabolism in plants**.

**Species**	**Gene**	**TFBS**	**Function**	**References**
*A. thaliana*	*At*MYB4	A(A/C)C(A/T)A (A/C)C	Associated with overproduction of *C4H*; cell wall biosynthesis; control sinaptate ester biosynthesis and provide UV stress protection	Jin et al., 2000; Dubos et al., 2010
	*At*MYB5	AACTAACT	Developmental regulation: Trichome morphogenesis and mucilage synthesis	Li et al., 2009
	*At*MYB11	AcCTACCa	Flavonol biosynthesis; activation of flavonol biosynthesis genes (*CHS, CHI, F3H, FLS*)	Stracke et al., 2007; Dubos et al., 2010; Pandey et al., 2015
	*At*MYB12	AcCTACCa	Enhance flavonol/chlorogenic acid content (regulated by bZIP TF under light stress)	
	*At*MYB14	*MREs*	Activates promoters of stilbene biosynthesis genes (*STS*); drought and salt tolerance	Höll et al., 2013
	*At*MYB15	*MREs*	Activates promoters of stilbene biosynthesis genes (*STS*); drought, cold and salt tolerance	Dubos et al., 2010; Höll et al., 2013
	*At*MYB21	*MREs, H-box, P-box*	Regulatory function: pollen and stamen maturation; regulates *PAL* gene	Davies and Schwinn, 2003; Cheng et al., 2009; Li and Laoke, 2016
	*At*MYB24	*MREs*	Regulatory function: pollen and stamen maturation	Cheng et al., 2009; Katiyar et al., 2012
	*At*MYB28		Associated with glucosinolates synthesis; response to herbivory	Gigolashvili et al., 2007
	*At*MYB29		Associated with glucosinolates synthesis; response to herbivory	Li and Laoke, 2016
	*At*MYB32		Regulates pollen wall composition; controls monolignol biosynthesis; enhances *DFR* and *ANS*; represses *COMT* gene	Preston et al., 2004; Dubos et al., 2010
	*At*MYB34		Associated with glucosinolates and auxin homeostasis; response to herbivory	Gigolashvili et al., 2007; Li and Laoke, 2016
	*At*MYB46		Under direct regulation of Secondary Wall-Associated NAC Domain Protein 1 (SND1), assists in secondary cell wall formation	Li and Laoke, 2016
	*At*MYB51		Associated with glucosinolates synthesis; response to herbivory	Li and Laoke, 2016
	*At*MYB54		Secondary wall synthesis and aids in lignification	Dubos et al., 2010; Liu et al., 2015; Li and Laoke, 2016
	*At*MYB57		Regulatory function: pollen and stamen maturation	Cheng et al., 2009; Li and Laoke, 2016
	*At*MYB58		Lignin synthesis; formation of secondary cell wall	Li and Laoke, 2016
	*At*MYB60		Transcriptional repressor of anthocyanin biosynthesis; ABA-mediated stomatal regulation	Dubos et al., 2010; Liu et al., 2015
	*At*MYB61	ACCTAC	Photomorphogenic control, mucilage deposition, stomatal aperture, xylem formation and carbon translocation to the roots; Regulates production of anthocyanin pigment-1	Li et al., 2009; Dubos et al., 2010; Prouse and Campbell, 2013; Liu et al., 2015; Li and Laoke, 2016
	*At*MYB63	*MREs*	Control anthocyanin biosynthesis in vegetative tissues by interacting with promoter AC elements	Dubos et al., 2010
	*At*MYB69		Secondary wall synthesis and aids in lignification	Dubos et al., 2010
	*At*MYB75/PAP1		Regulates production of anthocyanin pigment-1, positive regulator of lignin biosynthesis	Dubos et al., 2010; Liu et al., 2015
	*At*MYB76		Associated with glucosinolates synthesis; response to herbivory	Li and Laoke, 2016
	*At*MYB83		Under direct regulation of Secondary Wall-Associated NAC Domain Protein 1 (SND1), assists in secondary cell wall formation; upregulates various lignin biosynthesis genes	Liu et al., 2015; Li and Laoke, 2016
	*At*MYB85		Regulates lignin biosynthesis in fiber cells/vessels	Dubos et al., 2010
	*At*MYB90/PAP2		Control anthocyanin biosynthesis in vegetative tissues by interacting with promoter AC elements	Dubos et al., 2010; Liu et al., 2015
	*At*MYB103		Cell wall thickening in fiber cells	Dubos et al., 2010
	*At*MYB108		Associated with glucosinolates synthesis; response to herbivory	Li and Laoke, 2016
	*At*MYB111	AcCTACCa	Flavonol biosynthesis; activation of flavonol biosynthesis genes (*CHS, CHI, F3H, FLS*)	Stracke et al., 2007; Dubos et al., 2010; Liu et al., 2015; Pandey et al., 2015
	*At*MYB113	*MREs*	Control anthocyanin biosynthesis in vegetative tissues by interacting with promoter AC elements; interacts with bHLH and WD40 proteins	Dubos et al., 2010; Katiyar et al., 2012; Liu et al., 2015
	*At*MYB114			Du et al., 2012; Katiyar et al., 2012
	*At*MYB122		Associated with glucosinolates synthesis; response to herbivory	Li and Laoke, 2016
	*At*MYB123/TT2		Proanthocyanidin biosynthesis	Dubos et al., 2010; Liu et al., 2015
Apple (*Malus domestica*)	*Md*MYB1		Synthesis of anthocyanins (red pigment) in peel	Liu et al., 2015
	*Md*MYB3			
	*Md*MYB6		Repressor of anthocyanin biosynthesis	Liu et al., 2015
	*Md*MYB10		Activates the synthesis of anthocyanins peel, flesh, and foliage	Liu et al., 2015
	*Md*MYB110a		Anthocyanin biosynthesis: Mediates red coloration of fruit cortex in later phase of fruit maturity	Liu et al., 2015
	*Md*MYBA		Synthesis of anthocyanins (red pigment) in peel	Liu et al., 2015
	*Mdo*MYB121		Environmental stress tolerance	Cao et al., 2013
Grapevine (*Vitis* spp.)	*Vv*MYBA1/A2		Controls last step of anthocyanin biosynthesis mediated by UDP-Glucose flavonoid 3-O-Glucosyltransferase (UFGT); control fruit color	Matus et al., 2008
	*Vv*MYBA3		Control anthocyanin biosynthesis in other grapevine tissues	Matus et al., 2008
	*Vv*MYB14		Activates promoters of stilbene biosynthesis genes (*STS*); drought and salt tolerance	Höll et al., 2013
	*Vv*MYB15		Activates promoters of stilbene biosynthesis genes (*STS*); drought and salt tolerance	Höll et al., 2013
*Epimedium sagittatum*	*Es*MYBF1		Strong activator of promoters of *F3H, FLS*, thereby regulating flavonol biosynthesis	Huang W. et al., 2016
	*Es*MYBA1		Activates the promoters of *DFR* and *ANS*	Huang et al., 2013

Interestingly, several MYB TFs have dual roles, like *At*MYB 52, *At*MYB 54, and *At*MYB 69 regulate lignin biosynthesis (specialized metabolism), simultaneously regulating xylan and cellulose biosynthesis (primary metabolism). Similarly, *At*MYB46 is also associated with lignification in fibers and vessel tissues, simultaneously regulating xylan and cellulose deposition in *A. thaliana*. Most notably, research evidence pointed that the *cis*-element *MBSIIG* was bound favorably by MYB59 as well as MYB111. While MYB59 has been known to regulate cell cycle and root growth, MYB111 binding to *MBSIIG* was found to regulate flavonoid biosynthesis along with MYB11 and MYB12. Further, this element was found to be highly overrepresented in the promoter regions of several genes belonging to primary and specialized metabolism (Franco-Zorrilla et al., 2014). It is also known that some MYBs (*At*MYB63, *At*MYB90, *At*MYB113 and *At*MYB114) bring about the transcriptional regulation *via* binding to the *AC* elements present upstream to the stress-responsive genes through synergistic interaction with bHLH and WD40 TFs. The promoter regions of several primary (*LEA14, CWIN, vInv 1, SUSY*) and specialized metabolism genes (*CHI, HCT, ANS, DFR, F3H, PAL, C3H, GST*) have characteristic presence of *AC*-rich elements in their promoter regions. It thus makes it evident that MYB TFs play a bridging role to link primary and specialized metabolism in plants. Table 3 presents a comprehensive overview of the MYB TFs and their role in regulating metabolic processes in various plant genera.

#### The AP2/ERF superfamily

The APETALA2 TF family was initially linked to developmental regulation in plants, like floral development, seed germination and yield regulation. This TF family is associated with a few other pleiotropic roles, like regulating stress tolerance *via* expression of genes involved in abiotic stress response, disease resistance and ethylene/jasmonic acid/salicylic acid response (Cui et al., 2016; Guo et al., 2016). Based on the number of AP2/ERF DNA binding domains they possess, the AP2/ERF family is further classified into four subfamilies, namely ERF, DREB (one AP2/ERF domain); AP2 (two AP2/ERF domains) and RAV (one AP2 and an additional B3 DNA binding domain; Licausi et al., 2013; Guo et al., 2016; Huang Z. et al., 2016). The ERF subfamily in *Arabidopsis* is regulated either *via* a phytohormone dependent (like Ethylene, JA, ABA, auxin, cytokinin and SA; Guo and Ecker, 2004; Arora, 2005; Cheng et al., 2013; Dey and Vlot, 2015) or independent manner (*via Ethylene Insensitive or EIN* genes, stress like wounding, etc., Guo and Ecker, 2004; Arora, 2005; Dey and Vlot, 2015). ERFs have the ability to distinctly bind to the *GCC box* and *DRE* elements (under abiotic and biotic stress; Cheng et al., 2013; Guo et al., 2016) and upregulate downstream genes, thus forming a crucial component of stress mitigation mechanisms in plants. The DREB subfamily also plays a crucial role in abiotic stress mitigation by binding to the *DRE/CRT* elements (Dehydration Responsive Element/ C-Repeat Element) present upstream to stress responsive genes (like *RD29A, COR15a*, etc.), leading to plant responses to abiotic stresses like cold, drought and salinity (Chinnusamy et al., 2010; Basu et al., 2014). Similar *cis*-element *LTRE* (Low temperature Responsive Element) was found to be involved in mitigating cold stress. The promoter regions of *Arabidopsis Cor15A* gene (encoding cold-regulated chloroplastic protein, principally involved in cold stress regulation) showed the characteristic presence of *DRE* elements, while polyamine synthesis gene, *SamDC* (specialized metabolism) in rice showed the presence of both *DREs* as well as *LTREs* in their promoters (Basu et al., 2014). Promoter analysis of principal abiotic stress responsive genes in *A. thaliana* (*COR15A, COR15B, KIN1, KIN2 RD29B, RD29A, RD29B, RD22, RAB18* and *COR47*) demonstrated an overrepresentation of *DREs*, which is favorably bound by DREB1A and DREB2A (Sakuma et al., 2006). Research reports highlight the involvement of ERF and DREB subfamily in simultaneously upregulating genes belonging to the primary (*esk1, LEA, CAB, AS, DXS*) and specialized metabolism (*DcPAL3, STR, TDC, D4H, CPR*) by binding to the *GCC box*es in their promoters. It can therefore be inferred that the AP2 family TFs are involved not only in imparting stress tolerance to plants, but also form a crucial molecular link among diverse metabolic pathways.

The RAV subfamily TFs are more involved in imparting biotic stress tolerance to the plants *via* activation of the *PR* genes (Woo et al., 2010; Fu et al., 2014). One of the RAV proteins, RAV1 is known to be involved in ABA signaling, where it increased ABA insensitivity of seeds during germination (Feng et al., 2014). Scientific reports indicate that the RAV TFBS are overrepresented in the promoter regions of primary (*Em genes (Em1* and *Em6), LEA, AS*) and specialized metabolism genes (GST, LOX, SamDC, Feng et al., 2014; Moran Lauter et al., 2014). The RAV family TFs are also involved in regulating several other allied processes, like regulating metal starvation tolerance and controlling senescence-related gene expression. In *A. thaliana*, the promoter regions of principal cold responsive genes *COL1* (*CONSTANS-like 1*) and *COR27* demonstrated the presence of certain sequences called as “*Evening elements* (*EE*) and *EE-like (EEL*) elements” which were amplified in the presence of *ABRE-like (ABREL)* motif. Three *ABREL* motifs, along with four *EE* motifs could induce the expression of cold-responsive genes *COL1* and *COR27* (Mikkelsen and Thomashow, 2009). AP2/ERF family TFs can therefore serve as the missing molecular link between primary and specialized metabolism in plants. Table 4 presents a detailed account of the AP2/ERF TF family. However, among the AP2/ERF TFs, not many reports highlight the role of AP2 subfamily in regulating crucial genes under stressed conditions and futuristic research needs to highlight more in this aspect.

**Table 4:** **APETALA2 family TFs and their recognition sequences**.

**S. N**.	**Sub family**	**TFs**	**Core sequence/TFBS**	**Primary metabolism genes**	**Specialized metabolism genes**	**References**
1	DREB (ERF subfamily)	DREB1/CBF, DREB2A, DREB1D, ORCA1	A/GCCGAC	*COR15a, COR78, esk1, LEA, CAB, AS, DXS*	*STR, TDC, D4H, CPR*	Xin and Browse, 2000; Agarwal et al., 2006; Sakuma et al., 2006; Lata and Prasad, 2011; Licausi et al., 2013; Yamada and Sato, 2013
2	ERF	ERF-I-V, ORCA2, ERF221, EIN3, CRF, RAP2.6, RAP2.12, RAP2.2	AGCCGCC	*AOX, PDC, ADH1*	*DcPAL3, PMT, QPT, ODC, QS, MPO*	Kimura et al., 2008; Yamada and Sato, 2013
3	RAV	RAV1, RAV3, TEM1	CAACA	*Em genes (Em1* and *Em6), LEA, AS*	*GST, LOX, SamDC*	Woo et al., 2010; Licausi et al., 2013; Feng et al., 2014; Moran Lauter et al., 2014

*esk1, eskimo1 gene; CAB, Chlorophyll a/b-binding protein; AS, Anthranilate synthase; DXS, D-1-deoxyxylulose 5-phosphate synthase; STR, Strictosidine synthase; TDC, tryptophan decarboxylase; D4H, desacetoxyvindoline 4-hydroxylase; CPR, cytochrome P450 reductase; PMT, putrescine N-methyltransferase; QPT, quinolinate phosphoribosyltransferase; AOX, aspartate oxidase; ODC, ornithine decarboxylase; QS, quinolinic acid synthase; MPO, N-methylputrescine oxidase; PDC, Pyruvate decarboxylase*.

There are several other additional TFBS, which bring about cold stress mitigation, like the *MYC binding sites, G-box* and *ABRE* (Maruyama et al., 2012). Reports also suggest that AP2/ERF TFs work in tandem with bZIPs and MYBs to bring about synergistic regulation of cold stress tolerance by controlling ABA mediated gene expression in *Arabidopsis* (Pandey et al., 2005; Xu et al., 2011). Therefore, it can be suggested that a network of TFs is involved in coregulating diverse stress-responsive genes, which potentially form the missing molecular link between primary and specialized metabolism genes under stressed conditions. Although the active role of AP2 TFs subfamily in upregulating primary and specialized metabolism genes is not fully uncovered, deeper insights into this area would present a promising prospective in interconnecting diverse metabolic pathways.

## Future prospective

Newer insights into the interrelationships among multiple metabolic pathways are important to realize the subtle interplay of biomolecules within plants, as well as between plants and their environment. Primary and specialized metabolism serve as the backbone for the production of several therapeutically significant metabolites *in planta* (Tohge et al., 2013). Though under stress conditions plants overproduce certain key therapeutic metabolites, however it might have a negative impact on plant yield and productivity (Caretto et al., 2015). Therefore, an attempt to study coregulation of primary and specialized metabolism genes under certain stress conditions could pave a way to enhance both plant productivity and plant-derived therapeutic compounds.

Conventional stress-mitigation programmes either focus on breeding to develop robust, stress-tolerant plants or using plant growth regulators (like salicylic acid, ascorbic acid, brassinolides, etc.) to provide momentary stress-mitigation effects. Genetic engineering techniques to impart abiotic stress-tolerance to plants have focused on engineering stress-responsive TF genes in order to bring about effective stress response (Mickelbart et al., 2015). The current knowledge of *cis*-elements and TF interactions that bring about simultaneous upregulation of primary and specialized metabolism genes would help in developing tolerance to wide range of environmental stresses. Therefore, by adopting *cis*-element and TF engineering, scientists can develop robust crop varieties with high therapeutic potential. However, exhaustive research needs to be carried out before putting this technology to practice.

## Conclusion

The link between primary and specialized metabolic pathways in plants has been an area of extensive research in the recent years with prime focus being laid on stress mitigation, increased plant yield and enhanced production of specialized metabolites. In the recent decades, rising population has contributed to increased levels of environmental stress, thereby plunging the overall crop productivity. However, it is also known that stress alters the biochemical fingerprint of plants, thereby enhancing the production of therapeutic metabolites like alkaloids, flavonoids, stillbenoids and phenylpropanoids. Scientific studies aimed at imparting stress tolerance focus mainly on enhancing production of specialized metabolites through genetic engineering approaches. However, not many studies highlight the significance of the molecular interface connecting primary and specialized metabolites under stress conditions. Since the precursors for the specialized metabolites originate from primary metabolism, much efforts are needed to unravel the cross talk between the two pathways at the molecular level.

In our review, we have presented a comprehensive analysis of the interplay between primary and specialized metabolism in plants under stressed conditions. Phyto-stress brings about a remarkable change in the metabolic profile of plants, wherein diverse primary and specialized metabolites are overproduced. Among the primary metabolites, the levels of sugars, sugar alcohols and amino acids were predominantly enhanced. On the other hand, VOCs, phenylpropanoids and alkaloids were mainly overproduced from among the specialized metabolites. The striking observation that diverse forms of stress leaves behind similar biomolecular patterns points at the molecular level regulation occurring between these metabolic processes. In the process of stress mitigation, plants are known to concurrently induce the expression of diverse stress-responsive genes belonging to primary metabolism, specialized metabolism and TFs. Since the principal mode of regulation of various genes occurs at the transcriptional level, the prime focus was laid on the *cis*-element and TF interactions that can simultaneously regulate primary and specialized metabolism genes. Upon spanning the immense literature available on *cis*-element profiles in the promoter regions of these genes in different plant systems, it could be inferred that a predictable pattern of *cis*-element-TF interactions (like bZIP TFs which recognize and bind to *ABRE, AREB, G-box, ABI* elements; WRKY TFs binding to their recognition sites *W-box*; MYB TFs binding to the *MREs* and AP2 TFs binding to *DRE* and *GCC boxes*) could be seen among primary, specialized as well as TF genes. Many of these TFs possessed pleiotropic roles, like developmental regulation, controlling senescence, physiological functioning, phenylpropanoid metabolism regulation, etc. Moreover, this pattern was observed among genes belonging to diverse metabolic pathways in different plant species also (*V. vinifera, S. tuberosum, S. lycopersicum, E. haichowensis. O. sativa, N. tabacum, C. sativus, R. hybrida, Populus* spp., *H. vulgare, Z. mays, S. liaotungensis, M. acuminate, C. melo, etc*.) This pattern of *cis*-element-TF interactions holds the key toward simultaneous upregulation of diverse genes. However, despite the immense genomic data available for several plants (genome sequence available for more than 60 plant species), scientific reports discretely attempt at elucidating the transcriptional regulatory mechanisms of either primary metabolism or specialized metabolism or TF genes. The immense prospective offered by simultaneous transcriptional regulation of primary and specialized metabolism genes toward achieving a two-tier objective of stress-tolerance as well as improved therapeutic values needs to be harnessed at a full potential, as it is still in a nascent stage.

## Author contributions

The authors have equally contributed to the manuscript. SS and MN wrote the article, while BS conceptualized the manuscript and corrected the same.

## Funding

This work was supported by Department of Biotechnology, Ministry of Science and Technology, Govt. of India [grant number BT/Bio-CARe/02/10078/2013-14].

### Conflict of interest statement

The authors declare that the research was conducted in the absence of any commercial or financial relationships that could be construed as a potential conflict of interest.
